# Depathologizing Queer Adults’ Dating App Use in Canada: Convergent Mixed Methods Study

**DOI:** 10.2196/72452

**Published:** 2025-07-23

**Authors:** Jad Sinno, Amaya Perez-Brumer, Paul A Shuper, Daniel Grace

**Affiliations:** 1 Division of Social and Behavioural Health Sciences Dalla Lana School of Public Health University of Toronto Toronto, ON Canada; 2 Institute for Mental Health Policy Research & Campbell Family Mental Health Research Institute Centre for Addiction and Mental Health Toronto, ON Canada; 3 Department of Psychiatry University of Toronto Toronto, ON Canada

**Keywords:** mental health, queer adults, dating apps, queer theory, reparative theory, mixed methods

## Abstract

**Background:**

Dating apps are virtual sociosexual networking platforms that facilitate varying social and sexual relationships and have considerably changed the way that many queer individuals form social, sexual, and romantic connections. Despite evidence that social media use can be associated with either diminished or improved mental health, few studies have explored the association between dating apps and mental health among queer adults.

**Objective:**

Using reparative theory and a transformative paradigm, this research sought to critically explore the association between dating apps and mental health among queer adults in Canada..

**Methods:**

We used a convergent mixed methods design comprising an online survey (N=250) and one-on-one interviews (subsample of n=22) among queer adults from across Canada. Participants were recruited using Grindr advertisements and selected for diverse identities. The survey and interview collected information on dating app use and mental health. A structural equation model assessed the association between dating app use and mental health symptoms and the mediating role of discrimination and community connectedness. Hybrid reflexive thematic analysis of interviews elucidated how power and marginalization are negotiated, resisted, and refused in everyday app use.

**Results:**

Participants used an average of 3.22 (SD 1.78) dating apps, most commonly for casual sex (208/249, 83.5%). Dating app use was associated with increased life satisfaction (β=0.31, 95% CI 0.32-1.12; *P*<.001) and self-esteem (β=0.21, 95% CI 0.04-0.38; *P=*.02) but not with depression (β=−0.16, 95% CI −0.33 to 0.02; *P=*.07) or anxiety (β=−0.11, 95% CI −0.45 to 0.10; *P=*.20). Discrimination and seeking social approval were associated with adverse mental health. Although seeking friendship was the least commonly reported motivation (98/249, 39.4%), interviewees described making friends unintentionally through intimate experiences. Increased community connection was associated with heightened life satisfaction (β=0.18, 95% CI 0.14-0.82; *P=*.01) and self-esteem (β=0.13, 95% CI 0.004-0.28; *P=*.04). Interviewees described managing negative impacts of use by adjusting expectations, using technological features to avoid unwanted interactions, and welcoming unexpected interactions in addition to their desired connections from use. Participant accounts of the inconsistent and evolving ways to use dating apps revealed the complex relationship between app use and well-being.

**Conclusions:**

Queer peoples use dating apps conscientiously, leveraging hope and serendipity to stumble upon novel and welcomed connections. Queer peoples use strategies to promote their well-being while navigating this threatening internet-based sociosexual space. The mixed methods approach provides nuance to the relationship between dating app use and well-being, underscoring the context-dependent and temporally dynamic association between them.

## Introduction

### Background

Dating apps have significantly changed how sexually and gender-diverse (hereinafter, *queer*) people connect and form social, sexual, and romantic relationships [1-5]. However, a survey of 200,000 Grindr users, the most popular dating app among queer men [6,7], revealed that 77% felt regret after use [8]. Dating apps are a possible new stressor that needs to be better understood to improve the mental health of queer people. Despite advances in queer rights [9,10], queer peoples in Canada continue to experience mental health disparities; the alarming rate of depression and anxiety among queer peoples is a significant public health concern [11-14]. Lifetime rates of mood disorders are up to 3 times higher among Canadian sexual minority groups than among their heterosexual counterparts [15]. Previous literature has linked symptoms of psychological distress among queer individuals to various sociocultural stressors [16]. Queer communities reproduce social hierarchies [17], and discrimination on queer dating apps is prevalent, including femmephobia, transphobia, racism, ableism, fatphobia, ageism, and HIV and AIDS stigma [18-21].

Social media use among queer peoples might be both a risk factor and a protective mechanism against depression [22]. Problematic smartphone use, including addiction, has been linked to depression and anxiety, but this depends on one’s motivations for using the smartphone [23]. For example, online technologies may promote positive mental health by providing access to a wider queer community and identity expression [2]. Despite widespread evidence linking social media and smartphones to mental health, research exploring how dating apps influence queer adults’ mental health, and particularly positive health, is limited.

Existing literature on queer dating apps has often pathologized apps and users, focusing on sexual health risk, negative mental health, and isolation and loneliness [7,24-26]. While research exploring positive mental health is less abundant [24,27], studies show that enhancing well-being among queer individuals includes fostering a strong sense of belonging, living authentically, examining sexuality and relationships, and being free from gender-specific roles [27-30]; virtual sociosexual spaces can provide these opportunities. Thus, there is a need for reimagined research exploring the social organization and power relations on queer dating apps to understand potential positive or negative implications for well-being. This mixed methods research combined structural equation modeling with in-depth qualitative analysis to critically explore the association between dating apps and mental health among queer adults in Canada.

### Research Paradigm and Theoretical Framework

This study was situated in a transformative paradigm, an activist-oriented perspective providing an overarching framework for addressing social justice [31]. Transformative research examines power, social justice, and cultural complexity [31,32]. Queer theories critique normativity and understand how individuals resist, transform, and enact their subject positions [33,34]. A reparative queer approach within a transformative paradigm shifts from traditional critical perspectives of identifying risk and oppression toward a hopeful, positive, and constructive critique of the social [35,36]. Reparative theories mobilize queer critique for positive change and growth, emphasizing hope as hermeneutic and paying attention to affect to bear witness to queer peoples’ survival through the mundane and everyday life. In this research, the reparative framework explored how queer peoples use dating apps for connection and health promotion while navigating rejection, discrimination, fetishization, and other stressors.

We refer to queer people collectively as *queer peoples*. This is an intentional political and theoretical framing that emphasizes the diversity and multiplicity of queer communities. We use this terminology to highlight that queer individuals are not a monolith but rather comprise a diversity of sexual orientations, gender identities, cultural backgrounds, and lived experiences that are dynamic and fluid across spatial and temporal contexts. This research sought to interrogate power relations while being careful not to make broad generalizations or claims about a universal or collective experience. Moreover, we refer to virtual sociosexual networking apps (commonly referred to as hookup apps) as *dating apps* to acknowledge the role of these apps in fostering alternative, varying, and meaningful social relationships beyond casual sex, such as emotional support or nonsexual relationships [37,38]. Furthermore, we agree with previous scholars who argue that the term *hookup apps* perpetuates stereotypes about queer peoples’ app use by focusing narrowly on casual sex, ignoring other forms of intimacy and connection that users seek, and hides these platforms’ role in shaping queer sociability and community practices [37,38].

### Quantitative Research Model and Hypotheses

**Figure 1:**
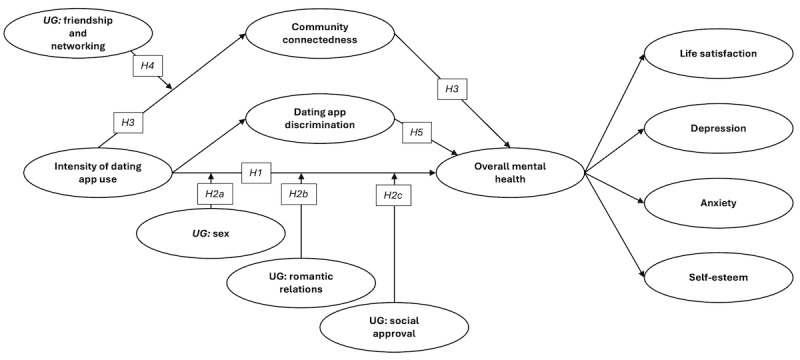
Hypothesized structural equation model based on existing literature. Overall mental health was conceptualized as a second-order latent variable. H: hypothesis; UG: Uses and Gratifications of Grindr scale.

The hypothesized research model for the quantitative strand of this study is shown in Figure 1.

#### Dating App Use and Mental Health Outcomes

Increased use of online technologies, including social media and dating apps, has been linked with negative consequences for users’ well-being. Studies reveal a positive correlation between social media use and symptoms of psychological distress, depression, and anxiety in general populations [39-42]. Similarly, the use of dating apps has been associated with social anxiety, depression, and poorer well-being among straight participants and queer men [43-45].

Hypothesis 1 is that greater *intensity of dating app use* will be negatively associated with *overall mental health*. Addiction to (and excessive use of) dating apps has been found to be likely related to motivations for use, such as self-esteem and sex-searching motives [46]. Queer men’s reasons for using dating apps include sex and hookups, romantic relationships, social approval or validation, friendship or networking, entertainment or passing time, and location-based searching [1,3,47,48]. A review of the literature suggests that the first 4 motives may influence the strength of the relationship outlined in hypothesis 1 [43,44].

Queer dating apps are designed for sex seeking, making them useful for casual sex [7,47,49]. There is preliminary quantitative evidence suggesting that queer men who use dating apps for sexual encounters report higher self-esteem and life satisfaction than those using them for other reasons [43]. An Australian mixed methods study (with a 73% straight sample) reported that the ease of finding sexual encounters on dating apps satisfied users’ sexual needs and increased feelings of competency and control over their sex lives [50]. Accordingly, it was expected that users seeking sex would not experience negative mental health associated with increased use.

Conversely, qualitative studies have reported that gay dating apps do not sufficiently facilitate long-term relationships because they are designed for casual sex, leading to user frustration [51,52]. Some queer men who used Grindr to find romantic relationships described being mistreated by others [7]. Similarly, Grindr users who could not find potential partners for a relationship discontinued their app use [53]. Some users reported ambivalence to others’ interest in long-term relationships due to the abundance of options on queer dating apps [53]. The highly sexualized nature of queer dating apps makes it harder for users seeking deeper connections to have successful interactions. Therefore, it was expected that users who sought romantic relationships on dating apps would report poorer overall mental health.

Surveys in general populations have reported that social media users can experience feelings of missing out or are continually making social comparisons, which are related to poorer health [54,55]. One study noted that, while some dating app users artificially bolster queer men’s perceived self-confidence by manipulating or curating their online personas, users struggle to maintain coherence between who they are on and off the app; the author concluded that these accounts evidenced a threat to psychological coherence [7]. Similarly, the strongest predictor of problematic Tinder use among a sample from the general population was using the app to enhance self-esteem [56]. Therefore, it was hypothesized that using dating apps for social inclusion or approval would increase the strength of the inverse relationship between dating app use and mental health.

Hence, hypothesis 2 is that the relationship between *intensity of dating app use* and *overall mental health* will be moderated by motivations for using dating apps (*sex* [hypothesis 2a], *relationships* [hypothesis 2b], and *social inclusion or approval* [hypothesis 2c]).

#### Dating App Use and Community Connection

Dating apps can provide queer peoples access to broader queer communities and a sense of belonging. Members of online communities have described in interviews greater ease connecting and relating to others compared to in-person interactions [57]. As such, dating apps can be socially empowering spaces facilitating meaningful interpersonal relations [2,7,57-59]. Frequent Grindr users who share personal information on the apps reported lower loneliness; however, users are less likely to share personal information when sexting or looking for casual sex [60]. Using Grindr facilitates new friendships and linkages with the gay community [1,47]. Zervoulis et al [43] reported an inverse relationship between dating app use and community connectedness among UK men who have sex with men. The authors suggested that this relationship was likely due to users’ motives, which could lead to differing outcomes.

Hence**,** greater *intensity of dating app use* will be negatively associated with *community connectedness*, which will be positively associated with *overall mental health* (hypothesis 3), and the relationship between *intensity of dating app use* and *community connectedness* will be moderated by the motive of *friendship or networking* (hypothesis 4).

#### Dating App Use and Discrimination

Queer users regularly report experiences of discrimination on dating apps. An interviewee in one study noted that the dating app Grindr often allowed the worst aspects of the gay community to flourish [3]. Various forms of discrimination have been reported on queer dating apps through qualitative research, including sex-obsessed shallowness, sexual racism, fatphobia (and discrimination against other body types), femmephobia, ageism, HIV discrimination, and general discrimination against physical attributes [3,18-21]. Hence, greater *intensity of dating app use* will be positively associated with dating app discrimination, which will be negatively associated with overall mental health (hypothesis 5).

## Methods

### Research Design

This study used a convergent mixed methods design with 2 strands: an online survey and individual interviews [61]. The survey explored the association between dating app use and mental health among queer dating app users. As studies exploring the relationship between dating app use and mental health have largely been quantitative, we incorporated a qualitative design to provide more nuance and explanation to associations between dating app use and well-being among our sample. Interviews provided in-depth accounts of queer adults’ experiences and perceptions of how dating apps influence their mental health. All aspects of the study were conducted virtually. A comprehensive summary of the methods has been reported elsewhere [62]. The methods and results are reported per the Strengthening the Reporting of Observational Studies in Epidemiology (STROBE) guidelines [63], the Checklist for Reporting Results of Internet E-Surveys (CHERRIES) [64] (Multimedia Appendix 1), and the COREQ (Consolidated Criteria for Reporting Qualitative Research) guidelines [65].

### Ethical Considerations

Ethics approval was obtained from the University of Toronto Research Ethics Board (RIS 42753). Informed consent was collected separately for each strand and documented electronically. Participants were informed that they needed to reach the end of the survey to obtain compensation information and were compensated with CAD $10 (US $7.27). Participants were compensated CAD $20 (US $14.54) for the interviews and CAD $10 (US $7.27) for the member check. Results have been deidentified or presented as aggregate data to maintain participants’ privacy.

### Participant Recruitment and Screening

#### Overview

Between October 2022 and February 2023, queer adults from across Canada were recruited using advertisements on Grindr [6]. Launched in 2009, Grindr is a location-based dating app primarily used by sexually diverse men and, until recently, was marketed primarily to this population [25]. A growing number of individuals who identify with diverse sexual and gender identities use Grindr, including transgender women, transgender men, and gender-nonconforming individuals [66,67]. The recruitment strategy for both strands was purposeful sampling with maximum variation [68]. Grindr users interested in the study were directed to an intake survey on the University of Toronto REDCap (Research Electronic Data Capture; Vanderbilt University) tool, which provided a brief overview of the research study and determined eligibility [69]. Participants had to identify as a sexual or gender diverse (queer) person, be aged ≥18 years, be currently living in Canada, speak English, and use dating apps. The lead author invited all prospective participants to a 5-minute Zoom call (Zoom Video Communications) to confirm eligibility and assigned participants to either the quantitative strand exclusively or both the quantitative and qualitative strands in keeping with the maximum variation approach [70]; the qualitative sample was nested within the quantitative sample [61]. Participants who completed the quantitative and qualitative strands were counterbalanced, meaning that half were assigned to complete the quantitative strand first to control for sequencing effects [71].

#### Quantitative Sample Size Considerations

The sample size calculation for the quantitative strand was based on the requirement to run a structural equation model (SEM) with 1 latent variable and 13 observed measures. The estimated range was 150 to 300 participants; recruitment continued until 250 participants completed survey responses, which aligns with the recommendation by Kline [72] for SEMs of 10 to 20 cases per model parameter and achieves 80% power with small to medium effect sizes [73,74].

#### Qualitative Sample Size Considerations

The recommended number of interviews to explore people’s experiences is 10 to 20 [75]. The concept of data saturation is inconsistent with our philosophical, theoretical, and analytic approach [75,76]. Our approach emphasizes that saturation—that is, the point at which collecting more data will no longer yield new insights—cannot be reached as individual experiences are complex, diverse, and unique. Instead, the research team aimed to conduct 20 interviews and assess whether enough data were collected to answer the research question of exploring power relationships on dating apps. More interviews were conducted as needed to collect additional information or include diverse voices and differing perspectives within the sample. This approach aligns with the concept of information power, which relies on researchers’ judgment regarding collecting sufficient data related to the purpose and goals of analysis [76].

### Quantitative Data Collection: Online Survey

#### Overview

A cross-sectional survey assessed the association between dating app use and mental health among queer adults. Participants received an email link to access the survey on Qualtrics (Qualtrics International Inc) and could complete it in more than one session (ie, they could save their responses and return to the survey later) [77].

The survey had approximately 200 questions about demographics, dating app use, and mental health. Most questions were quantitative (eg, multiple choice and Likert scales), but some included open-ended or free-form responses; 3 questions at the beginning, middle, and end of the survey allowed participants to freely elaborate on their responses or leave comments. Adaptive questioning displayed subsequent questions based on previous responses, and participants could skip questions they did not wish to answer and return to previous sections to revise their responses. In total, 3 attention checks were placed throughout the survey (eg, “Even if these instructions seem strange to you, please pick the response option ‘Disagree’”); participants who failed 2/3 attention checks were excluded from the analytic sample.

#### Sociodemographic and Mental Health Characteristics

Age, gender identity, sex at birth, sexual orientation, relationship status, HIV status, citizenship status, cultural identity, ancestry, educational level, employment status, income, location in Canada, and financial well-being (abbreviated version of the Consumer Financial Protection Bureau Financial Well-Being Scale [78]) were collected. Participants could describe their gender identity, sexual orientation, and cultural identity in free-form text. Ancestry (ie, ethno-racial origins of one’s ancestors) and cultural identity (ie, the self-defined sense of belonging to a group, including but not limited to ethno-racial identity) were defined and asked separately; ancestry was a multiple-choice question. Participants were also asked to report any mental health diagnoses and self-rate their overall mental health from *poor* to *excellent*.

#### Dating App Use

Participants were asked about their dating app use. They reported the duration of app use and the dating apps they had used for at least a week in the previous 12 months. In total, 2 questions adapted from the Grindr Use Characteristics and Behaviors questionnaire asked how often dating apps were used and the reason for using them [26]; “Grindr” was replaced with “dating apps” to ask participants about dating apps generally. Participants were asked to report their current and initial reasons for using dating apps, as well as their top current and initial reasons.

#### Intensity of Dating App Use

Initially developed by Ellison et al [79], the Facebook Intensity Scale was later amended to the Grindr Intensity Scale and then to the Gay Dating Apps Intensity Scale [43,60]. “Gay dating apps” was replaced with “dating apps” given that many dating apps are not exclusively used by gay people (eg, Tinder or Bumble). The questionnaire comprised 6 questions measuring the intensity of dating app use from “strongly disagree” to “strongly agree” on a 5-point Likert scale. The Cronbach α in this study was 0.71.

#### Motivations for Using Dating Apps

The Uses and Gratifications of Grindr scale assesses users’ physical, social, and psychological gratifications when using Grindr [3]. “Grindr” was replaced with “dating apps.” Language such as “guys,” “men,” and “gay” were replaced with “users,” “people,” and “queer” where appropriate to include all gender identities. Responses were measured on a 7-point Likert scale from “not at all important” to “extremely important.” There are 6 subscales, each related to a different motivation for app use. The overall scale had a Cronbach α of 0.89, with values for the subscales ranging from 0.61 to 0.89.

#### Dating App Discrimination

The Everyday Discrimination Scale captures self-reports of interpersonal discrimination and includes 9 items measured on a 6-point Likert scale from “never” to “almost every day” [80,81]. The scale prompt was amended to the following: “When interacting with others on dating apps, how often do the following things happen to you?” The item related to restaurants and stores was omitted. Participants were also asked to choose from a multiple-choice list what they believed to be the reasons for their experience of discrimination. The Cronbach α was 0.88.

#### Community Connectedness

The Psychological Sense of LGBT Community Scale measures queer peoples’ sense of belonging to the wider queer community [82]. The scale includes 22 items on a 5-point Likert scale from “none” to “a great deal.” The total score of the items determines participants’ psychological sense of community connectedness and belonging [43]; higher scores indicate a greater community connectedness. The Cronbach α was 0.88.

#### Life Satisfaction

The Satisfaction With Life Scale comprises assessing participants’ perceptions of their subjective well-being [83,84]. The items are rated on a 7-point Likert scale from “strongly disagree” to “strongly agree,” with higher scores indicating greater perceived life satisfaction. The Cronbach α was 0.90.

#### Depression

The Center for Epidemiological Studies Depression Scale includes 10 items rated on a 4-point Likert scale about participants’ feelings and actions in the previous week (7 days) [85-87]. Higher scores indicate greater depressive symptoms. The Cronbach α was 0.86.

#### Anxiety

The Patient-Reported Outcomes Measurement Information System Anxiety scale short form is an 8-item questionnaire rated on a 5-point Likert scale (from “Never” to “Always”). Participants were asked how often they felt as stated in each statement in the previous 7 days. Higher scores indicate higher anxiety symptoms [88,89]. The Cronbach α was 0.95.

#### Self-Esteem

The 10-item Rosenberg Self-Esteem Scale measures self-worth and global self-esteem [90,91]. The questionnaire is measured on a 4-point Likert scale from “strongly disagree” to “strongly agree,” with higher scores indicating higher self-esteem. The Cronbach α was 0.91.

### Quantitative Data Analysis: Structural Equation Modeling

Descriptive statistics were calculated in SPSS (version 28; IBM Corp) [92]. The hypothesized SEM was analyzed using *lavaan* in R (R Foundation for Statistical Computing) [93,94]. Confirmatory factor analyses were first completed to assess fit indices for all scales. In the SEM, overall well-being was a second-order latent variable comprising life satisfaction, depression, anxiety, and self-esteem. Model fit was assessed using a comparative fit index (CFI) of >0.95, a Tucker-Lewis index (TLI) of >0.90, a root mean square error of approximation (RMSEA) of <0.08 (90% CI not exceeding 0.08), and a standardized root mean square residual of <0.08 [72,95]. The residual data were screened for violations of normality and outliers. A maximum likelihood estimator was used to adjust for any nonnormality [96]. Missing data were handled using full-information maximum likelihood [97].

The SEM used a single-indicator latent variable approach for all variables, where observed scores are used as a single indicator of latent variables while accounting for measurement error by specifying a score for reliability; scale means were used as single indicators, and the Cronbach α accounted for measurement error [72,98]. This approach allows for conceptual and methodological consistency of the latent constructs measured in this research and improved fit of complex models as compared to models with observed variables. Nested model comparison was conducted hierarchically using the Akaike information criterion and sample size–adjusted Bayes information criterion; lower scores indicated a better model [72,99]. Model 1 excluded interaction terms and overall mental health as a second-order latent variable. This was compared to model 2, which included overall mental health without interaction terms. The better model was then compared to model 3, which included interaction terms. The best model was interpreted and reported. Path coefficients are described as strong if β≥|0.30|, as moderate if β≥|0.20|, and as small if β≥|0.10| [72].

### Qualitative Data Collection: Interviews

One-on-one semistructured interviews were conducted via Zoom and audio recorded [70,75]. The lead author, a queer nonbinary dating app user and doctoral candidate at the time of data collection, conducted the interviews. The interview guide was emailed to participants ahead of their scheduled interviews and comprised 9 topics and domains broadly encompassing personal dating app use, self-presentation, interactions, feelings, impact on well-being and identity, impact on queer communities, and dating app features and platforms. Sessions lasted between 38 and 84 minutes, averaging approximately 59 (SD 13.36) minutes.

### Qualitative Data Analysis: Hybrid Thematic Analysis

The audio recordings were transcribed using the artificial intelligence transcription software Grain (Grain Intelligence Inc) [100]. The lead author reviewed each transcript alongside the recording for accuracy. Transcripts were analyzed in NVivo (version 14; QSR International) using reflexive and hybrid thematic analysis informed by reparative theory [101-103]. Hybrid thematic analysis combines data-driven and theory-driven analyses. Familiarization with the dataset involved interviewing, listening to recordings, and rereading the 3 most extensive transcripts to develop the initial coding structure. Semantic coding of the entire dataset generated initial themes. Research notes were documented during interviews, transcript revision, and coding; these informed the analysis process. A member check was completed (see the Mixed Methods Integration and Member Checking section).

Following the member check, the lead author recoded the dataset conceptually and latently, as well as deductively through a reparative lens. The analysis paid close attention to the feelings and intimacies of experience, uncovering how participants collect the resources they need to survive in the threatening world [104]. Quotes from interviewees are followed with an ID (eg, P12).

### Measures of Rigor for Qualitative Data

To improve qualitative rigor, we have reported the results according to the COREQ checklist [65]. Moreover, the research team prioritized ongoing reflexivity, where the lead researcher reflected on their lived experience and how it impacts the research process; we emphasize that the results have been informed by the lived experience of the lead researcher as a queer adult who uses dating apps and this has contributed to the interpretation of the findings. The interview guide was reviewed by other members of the research team and piloted with 10 queer individuals who had used dating apps. The first few interviews helped determine whether the interview guide needed revision, but this was not the case. Participant quotes have been assigned a nonidentifiable participant number that allows readers to determine whether the supporting quotes were said by the same or a different participant. Finally, a responsible approach to qualitative research and member checking that is consistent with a transformative paradigm was incorporated throughout the research process. For example, participants were able to explain or discuss thoroughly the topics they wanted, and the interviewer confirmed understanding of participants’ experiences by repeating them to participants [105]. A final member check after interpretation also took place (see the Mixed Methods Integration and Member Checking section).

### Mixed Methods Integration and Member Checking

This research study included several points of integration between strands [61,106]. A nested sample collected queer adults’ dating app experiences and health needs. Parallel data collection and analysis were compared, looking for confirmation, expansion, or discordance between sources [107]. A summary report and a responsible approach to member checking was completed between January 2024 and February 2024; the 22 participants who completed both strands were invited to review initial findings and the research team’s interpretation of the data [105]. The member check process included emailing the summary report to participants alongside questions about the interpretation and accuracy of the research findings, how participants felt about these findings, and how they hoped that these findings would be used in the future; a full detail of this process has been reported elsewhere [62]. The findings were contextualized by the research team’s philosophical and theoretical commitments to interrogate power relations on dating apps for social justice and transformation. The findings are reported using a weaving approach [61].

## Results

### Sociodemographic Characteristics

**Figure 2:**
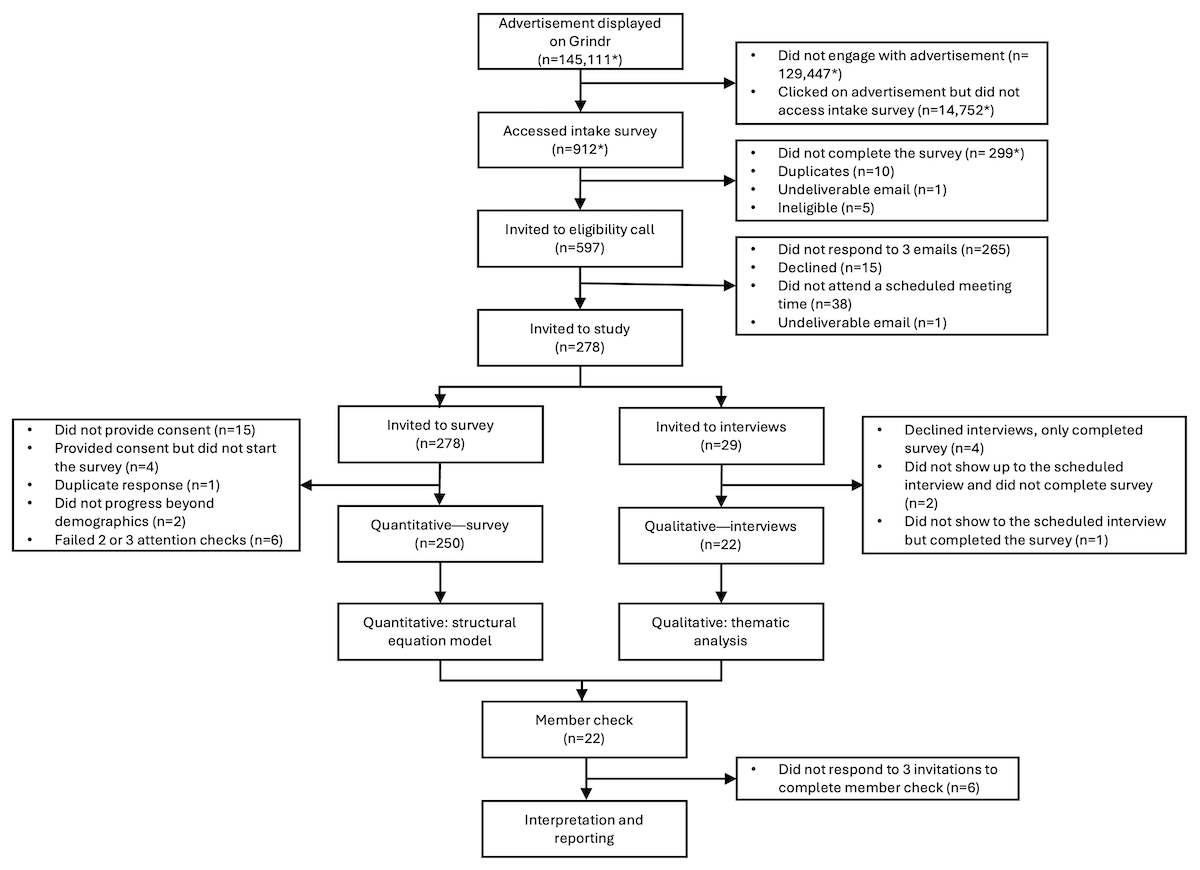
Flowchart of participant screening and research process. The diagram shows the convergent mixed methods research design. *Numbers do not necessarily reflect unique individuals.

Figure 2 shows the recruitment process. The completion rate among individuals who consented to participate in the quantitative strand was 97.7% (256/262). The final analytic sample for the online survey was 250 participants across Canada—the sociodemographic characteristics of the survey participants are listed in Multimedia Appendix 2. The mean age was 36.18 (SD 12.39) years, ranging from 18 to 71 years. Most participants were assigned male at birth (242/250, 96.8%) and identified as cisgender men (200/250, 80%) and gay (167/250, 66.8%). Half (129/250, 51.6%) reported good, very good, or excellent mental health, and two-thirds (164/248, 66.1%) self-reported at least one mental health diagnosis.

A subsample of 22 participants was interviewed, 12 (55%) of whom completed the survey first (Table 1). Their mean age was 32.6 (SD 10.0) years, ranging from 19 to 63 years. Half (11/22, 50%) identified as a men, cisgender men, or male, and approximately a third (8/22, 36%) identified as gay. Interviewees represented a diversity of cultural identities and locations in Canada. A total of 64% (14/22) of the interviewees completed the member check.

**Table 1:** Demographic characteristics of the intervieweesa.

ID	Age (y)	Gender	Sexual orientation	Relationship status	Cultural identity	Location	Population of town or city	Component completed first
1	19	Cis man	Bisexual	Single	Ukrainian	Alberta	500,000-1,499,999	Interview^b^
2	19	Trans woman	Lesbian	Single	Canadian	British Columbia	10,000-49,999	Survey^b^
3	25	Nonbinary	Pansexual	Single	Canadian; Albertan	Alberta	500,000-1,499,999	Survey^b^
4	25	Figuring it out	Homoflexible	Single	African; West African; Nigerian	Eastern Ontario	50,000-199,999	Survey^b^
5	27	Man	Bisexual	Single	Quebecois and Métis	Metropolitan Montréal	≥1,500,000	Survey
6	28	Cis man	Gay	Single	Canadian	British Columbia	10,000-49,999	Interview^b^
7	29	Trans woman	Bisexual	Single	Colombian	Central Ontario	500,000-1,499,999	Interview
8	29	Man	Gay	Relationship	Canadian; Hongkonger	Central Ontario	200,000-499,999	Interview
9	29	Genderqueer^c^	Queer	Single	Quebecois; Black	British Columbia	0-9999	Survey^b^
10	30	Trans woman	Pansexual	Single	Latin	Central Ontario	0-9999	Survey
11	30	Nonbinary	Gay	Relationship	English-speaking Canadian	Metropolitan Montréal	≥1,500,000	Survey^b^
12	31	Cis man	Gay	Single	Black	Western Quebec	50,000-199,999	Survey^b^
13	31	Male^d^	Queer; gay; pansexual	Polyamorous	Canadian, English, Scottish, Irish, Franco-Ontarian, Indigenous, pagan, heathen, and anglophone	Southwestern Ontario	200,000-499,999	Interview^b^
14	32	Bigender	Pansexual	Single	Canadian	Central Ontario	50,000-199,999	Interview^b^
15	32	Male	Gay	Single	Asian	Metropolitan Toronto	≥1,500,000	Survey^b^
16	33	Man	Bisexual	Single	White	Metropolitan Toronto	≥1,500,000	Interview
17	33	Woman	Pansexual	Single	Canadian; Cascadian	British Columbia	50,000-199,999	Survey
18	37	Nonbinary^d^	Raging queer	Single	Mixed race; Chinese-Canadian	Alberta	500,000-1,499,999	Survey^b^
19	40	Man	Gay	Single	Middle Eastern–Canadian	Alberta	500,000-1,499,999	Survey^b^
20	44	Woman	Pansexual	Single	Canadian-Australian	Alberta	500,000-1,499,999	Interview
21	52	Male	Gay	Single	Islander—Prince Edward Islander, Canadian, English, and Presbyterian	Nova Scotia	0-9999	Interview
22	63	Man	Gay	Single	Chinese-Canadian	Metropolitan Toronto	≥1,500,000	Interview^b^

^a^Demographic information for the interviewees was collected through survey responses. Gender, sexual orientation, and cultural identity allowed for open-ended responses; participants’ exact verbiage is reported in the table. Location was recoded from the first digit of participants’ postal code forward station area. All participants were assigned male at birth unless otherwise specified.

^b^Completed member check.

^c^Assigned intersex at birth.

^d^Assigned female at birth.

### Queer Adults’ Dating App Use

The survey respondents’ dating app use characteristics are summarized in Multimedia Appendix 3. The mean number of dating apps used per participant was 3.22 (SD 1.78; range 1-11); 85.2% (213/250) of the participants reported using ≥2 apps in the previous 12 months. Grindr (248/250, 99.2%), Tinder (120/250, 48%), and Scruff (95/250, 38%) were the most used for at least 1 week in the previous year, with the overwhelming majority using dating apps daily (209/250, 83.6%) and for more than a year (215/249, 86.3%). Approximately half of the participants’ top reason for using dating apps was to find sexual partners, both when they started using dating apps (119/243, 49%) and currently (121/248, 48.8%). However, participants reported, on average, 2.89 (SD 1.33; range 1-6) reasons for using dating apps, indicating that, generally, participants used dating apps for several reasons simultaneously. The mean score on the sex subscale of the Uses and Gratifications of Grindr scale was the highest (5.11, SD 1.35), indicating that, on average, using dating apps for sex was moderately important to participants. These findings reflect the multivalent and differing uses of dating apps fostered hope and serendipity among the participants, which are described in the first theme: *the contradictory, messy, and inconsistent use of dating apps*.

Interview participants described various ways in which they used dating apps, often *messily*, inconsistently, and evolving with their changing needs—*messy* insofar as their app use was nonlinear, fluid, and sometimes contradictory, with intentions and outcomes not always aligned. Participants typically used dating apps for multiple reasons or in multiple ways. Some described clear goals for their use but were open to serendipity and unexpected relationships. A participant explained the following:

Sometimes, I’m just happy to go instantly grab a coffee with someone. So, it’s kind of like that’s what I mean about not really having a specific goal...I take my time, get to know people, but it kind of is what it is.

P14

P14 described their dating app use as somewhat mundane and open ended. They only categorized their use when specifically probed about their goals:

Primarily, I guess my goal is romantic...and then probably sexual after that and then friends after that. But I’ve probably made more friends than anything else. So, the lowest tier is the highest results.

P14

Participants’ dating app use also fluctuated over time, with periods of heightened use and breaks during which use was suspended entirely. One participant explained the following:

My interest in Grindr goes up, it spikes up when I’m looking for sexual partners, and then it goes like this [gestures downward]...I definitely lose interest at a gradual rate over time, and then I go from checking it multiple times a day to once a day to once every few days.

P3

Participants described their experience with dating apps positively (as affirming, improving their quality of life, and leading to making new connections) and negatively (as isolating, distressing, frustrating, and addictive). Their accounts indicate the complexity and nuance of online dating experiences, where similar instances may be both positive and negative (eg, blocking as a safety mechanism but being blocked as isolating), be temporally or contextually dependent (eg, the inability to make connections at low periods in one’s life is especially frustrating and disheartening), or have different implications for different users (eg, different users have different capacities or expectations of the online space). Participants’ satisfaction with dating apps differed according to the connections they hoped to make. Many noted that arranging casual sexual encounters was easily and readily accessible. Accordingly, participants who primarily used dating apps for sex found satisfaction because they were often able to make sexual arrangements. However, others looking for platonic or romantic connections noted that they may not be successful. They also acknowledged that long-term friendships or romantic relationships are challenging to foster. Some users adjusted their expectations, recognizing that interactions were often short-lived and rejection was common.

The contradictory nature of social connection online felt frustrating to some users. For example, ghosting (abrupt ending of communication without explanation), blocking, and ignoring are commonly used to communicate rejection (instead of explicitly rejecting another user) but contribute to feelings of isolation. One participant explained the following:

What I find strange is how people mostly ghost, which make the experience of the app lonely. And I find it strange to be on the app looking at people that do not answer to me and find them every day on the same grid.

P12

However, participants also described how they used ghosting and ignoring to communicate disinterest and avoid unsafe or unwelcome interactions.

Dating apps also offer the illusion of endless potential connections, encouraging users to continually seek new matches while simultaneously connecting with others. This illusion contributes to the addictiveness of dating apps; the next attempt to connect with someone may be successful (and perhaps better than a current connection). Nevertheless, several participants explained that having access to (potentially) an endless number of possible connections was a valuable feature, which is why they used dating apps:

The convenience is the range of contacts, range of people, the diversity. Basically, it is a community on one app.... It’s a window to a world and also for people to see me.

P22

Despite some participants describing their experiences as frustrating, they continued to use dating apps to expand their options. As the following participant explained, they hoped that their dating app use would facilitate fostering new relationships:

I’ve actually deleted most [dating apps] a number of times now, but why I continue to use them is, I also try to meet people in person, like all the old-fashioned way, and that hasn’t really worked either. So, I’m just trying to keep my options open. It’s very frustrating. Very depressing at times...I think I mostly continue trying to use them for lack of a better alternative.

P20

The hope of making the desired connections online was a reason to continue using them despite a history of unsuccessful use.

### Confirmatory Factor Analyses and SEM Selection

Confirmatory factor analyses demonstrated acceptable fit indexes for intensity of dating app use, motivations for using dating apps, dating app discrimination, community connectedness, life satisfaction, depression, anxiety, and self-esteem (Table 2). Factor loadings were significant at *P*<.05 for all scales. Screening the residuals revealed mild violations of normality that were acceptable, so a maximum likelihood estimator was used. No outliers were apparent.

**Table 2:** Confirmatory factor analysis goodness-of-fit indexes^a^.

	Chi-square (*df*)	CFI^b^	TLI^c^	RMSEA^d^	SRMR^e^
Motivations for dating app use	207.7 (98)	0.95	0.93	0.07	0.07
Dating app use intensity	29.1 (9)	0.94	0.90	0.10	0.06
Dating app discrimination	66.3 (14)	0.93	0.89	0.13	0.05
Community connectedness	57.5 (20)	0.95	0.92	0.09	0.05
Life satisfaction	3.2 (5)	1.00	1.00	<0.01	0.01
Depression	98.7 (27)	0.90	0.87	0.11	0.06
Anxiety	49.4 (20)	0.98	0.97	0.09	0.02
Self-esteem	127.8 (35)	0.92	0.89	0.11	0.05

^a^Robust estimates were used.

^b^CFI: comparative fit index.

^c^TLI: Tucker-Lewis index.

^d^RMSEA: root mean square error of approximation.

^e^SRMR: standardized root mean square residual.

**Figure 3:**
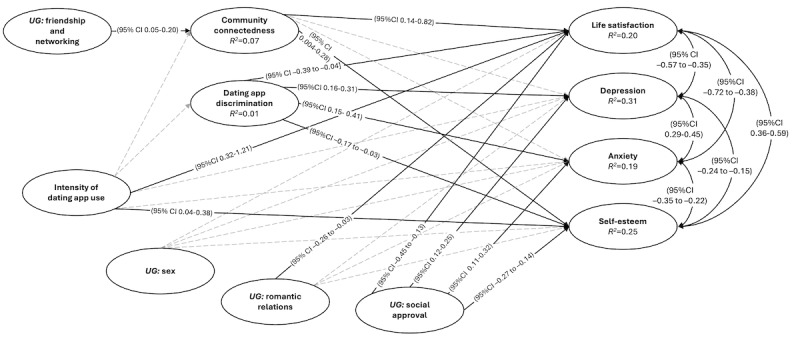
Results of structural equation model 1 with single-indicator latent variables. The 95% CIs of intervals for paths not including 0 are reported. UG: Uses and Gratifications of Grindr scale.

Table 3 summarizes the nested model comparison. Model 1, comprising single-indicator latent variables without overall mental health and interaction terms, performed better than models 2 and 3; the Akaike information criterion and sample size–adjusted Bayes information criterion indicate very strong support for selecting model 1 [99]. The SEM fit well (χ^2^_12_=91.8; comparative fit index=0.98; Tucker-Lewis index=0.92; RMSEA=0.07, 90% CI 0.04-0.11; standardized root mean square residual=0.04), although it failed to meet the 90% CI cutoff for the RMSEA excluding values of >0.08. The pathway coefficients are summarized in Table 4, and model 1 is shown in Figure 3.

**Table 3:** Structural equation model goodness-of-fit indexes—nested model comparison^a^.

	AIC^b^	ΔAIC^c^	SABIC^d^	ΔSABIC	Chi-square (*df*)	CFI^e^	TLI^f^	RMSEA^g^ (90% CI)	SRMR^h^
Model 1	7224.5	—^i^	7247.3	—	27.3 (12)	0.98	0.92	0.07 (0.04-0.11)	0.04
Model 2	7277.7	53.2	7293.5	46.2	117.7 (32)	0.90	0.83	0.11 (0.08-0.13)	0.06
Model 3	7269.1	44.6	7287.7	40.4	91.8 (24)	0.92	0.81	0.11 (0.09-0.13)	0.10

^a^Model 1 only included single-indicator latent variables without a second-order latent variable for overall mental health or interaction terms. Model 2 was the same as model 1 with overall mental health included as a second-order latent variable. Model 3 was the same as model 1 with interaction terms added. Robust estimates were used.

^b^AIC: Akaike information criterion.

^c^Indicates the difference between the corresponding model and model 1.

^d^SABIC: sample size–adjusted Bayes information criterion.

^e^CFI: comparative fit index.

^f^TLI: Tucker-Lewis index.

^g^RMSEA: root mean square error of approximation.

^h^SRMR: standardized root mean square residual.

^i^Not applicable.

**Table 4:** Pathway coefficients of structural equation model 1.

Pathways	B^a^ (95% CI)	β^b^	SE	*P* value
Dating app use intensity→life satisfaction	0.72 (0.32 to 1.12)	0.31	0.21	<.001
Dating app discrimination→life satisfaction	−0.21 (−0.39 to −0.04)	−0.15	0.09	.02
Community connectedness→life satisfaction	0.48 (0.14 to 0.82)	0.18	0.18	.01
Social approval→life satisfaction	−0.29 (−0.45 to −0.13)	−0.29	0.08	<.001
Sex→life satisfaction	0.03 (−0.18 to 0.24)	0.03	0.11	.78
Romance→life satisfaction	−0.15 (−0.26 to −0.03)	−0.18	0.06	.01
Dating app use intensity→depression	−0.16 (−0.33 to 0.02)	−0.16	0.09	.07
Dating app discrimination→depression	0.24 (0.16 to 0.31)	0.39	0.04	<.001
Community connectedness→depression	0.00 (−0.15 to 0.14)	0.00	0.07	.97
Social approval→depression	0.18 (0.12 to 0.25)	0.42	0.03	<.001
Sex→depression	−0.07 (−0.15 to 0.02)	−0.14	0.04	.11
Romance→depression	0.02 (−0.03 to 0.07)	0.05	0.03	.46
Dating app use intensity→anxiety	−0.18 (−0.45 to 0.10)	−0.11	0.14	.20
Dating app discrimination→anxiety	0.28 (0.15 to 0.41)	0.30	0.07	<.001
Community connectedness→anxiety	0.02 (−0.21 to 0.26)	0.01	0.12	.85
Social approval→anxiety	0.22 (0.11 to 0.32)	0.33	0.05	<.001
Sex→anxiety	−0.02 (−0.16 to 0.11)	−0.03	0.07	.72
Romance→anxiety	0.04 (−0.05 to 0.12)	0.07	0.04	.39
Dating app use intensity→self-esteem	0.21 (0.04 to 0.38)	0.21	0.09	.02
Dating app discrimination→self-esteem	−0.10 (−0.17 to −0.03)	−0.17	0.04	.01
Community connectedness→self-esteem	0.14 (0.004 to 0.28)	0.13	0.07	.04
Social approval→self-esteem	−0.21 (−0.27 to −0.14)	−0.50	0.03	<.001
Sex→self-esteem	0.05 (−0.03 to 0.13)	0.11	0.04	.24
Romance→self-esteem	−0.01 (−0.06 to 0.04)	−0.03	0.03	.66
Dating app use intensity→community connectedness	0.00 (−0.15 to 0.15)	0.00	0.08	.99
Friendship→community connectedness	0.13 (0.05 to 0.20)	0.26	0.04	<.001
Dating app use intensity→dating app discrimination	0.18 (−0.11 to 0.47)	0.11	0.15	.23

^a^Unstandardized coefficient.

^b^Standardized coefficient.

### Dating App Use and Mental Health

The SEM revealed that the intensity of dating app use was strongly associated with life satisfaction (β=0.31; *P*<.001) and moderately associated with self-esteem (β=0.21; *P=*.02); the more intense the use of dating apps, the greater the life satisfaction and self-esteem of the participants. The relationship between dating app use intensity and depression (β=−0.16; *P=*.07) as well as anxiety (β=−0.11; *P=*.20) trended toward being inversely related, but these associations were small and likely negligible, particularly for anxiety.

Regarding motivations, seeking sex was not associated with any mental health variables. There was a small to moderate negative relationship between seeking romance and life satisfaction (β=−0.18; *P=*.01) but there was no relationship between seeking romance and depression, anxiety, or self-esteem. Seeking social approval was negatively associated with life satisfaction (β=−0.29; *P*<.001) and self-esteem (β=−0.50; *P*<.001) but positively associated with depression (β=0.42; *P*<.001) and anxiety (β=0.33; *P*<.001); that is, using dating apps for external validation was associated with poorer mental health. These quantitative findings offer positive associations with dating app use, complicating narratives of pathology and psychological distress commonly associated with these apps; they are further explored in the second theme, *the perceived impact of dating apps on well-being and use of coping mechanisms as health promotion*.

Many interview participants responded neutrally about their dating app use and its impact on their well-being. Dating apps were just another tool for social connection, similar to other social media (eg, TikTok and Instagram). For them, dating apps did not meaningfully promote or diminish their well-being. Many described strategies to make the best of their experience online. One participant described the mundane nature of their app use:

I would say it’s like a peaks and valleys kind of thing...I would say it’s mostly just a neutral kind of thing to me. I could delete them all and I don’t think it would affect me that much, by using them, I don’t feel it. In the past, definitely I struggled a lot, just with people’s opinions and my self-esteem kind of thing on it. But I have been on dating apps for like several years now. So, I guess maybe I’ve either built up a tougher skin or just kind of realize more that they’re not like just a success factory, if that makes sense.

P17

Other participants similarly acknowledged that dating apps can promote health and also negatively impact their well-being. One participant explained that using dating apps encouraged them to work out and be active, not necessarily for the goal of health but rather to enhance body image:

[Dating apps] kind of makes me want to go and work out more instead of just like saying I don’t feel like it. Actively go and follow the things, like the routines I want to follow, to be able to have the appearance that I want to have. Because it's been a long time committing to that appearance. So, it's like telling me that there is a reward for those, uh, kind of efforts, for following that effort and following that discipline. It's like, okay, do you see that you are getting the attention that you wanted? Well, keep doing what you have to do.

P7

Among participants who described dating apps as negatively impacting their well-being, many noted that rejection and fleeting connections online were the reasons why they experienced adverse mental health. One transgender woman explained the following:

I would say [dating apps] affects [my mental health] negatively. Most of the time it’s disappointment. If you actually do make a quick connection, then it just leads to disappointment. And, uh, a lot of the time if I see someone else I’m genuinely interested in, especially with non-Grindr apps, where you typically have if you’re using it for free, you have to match before you can chat...a lot of the time, it’s just you’ll never match back. It’s like, oh, this person actually looks really cool. And for instance, they might say they’re a trans ally and all this stuff. It’s like, well, surely, we could be at least friends, but nothing ever happens. And so, I think as far as mental health goes, I find it unhealthy. It’s not conducive to good feelings of self-worth or self-esteem.

P20

Another participant similarly explained that, when they started using dating apps to seek external validation, it felt good initially but then had a negative impact on their well-being:

So, I started using [dating apps] for validation. And like it did when I quit addiction, that feels good for a short period of time, but then you kind of just feel gross about the interactions you’ve had. And then, unfortunately, due to my financial situation and the fact that I was financially dependent on my family that wasn’t very supportive, I had to start using Grindr to make money, which is a very low point in my life, and it didn’t make me feel all that great.

P2

This participant’s negative experience with dating apps was further compounded by a lack of social support and financial precarity, contributing to poorer well-being; the negative impact of dating app use was dependent on their social and financial conditions at the time.

Participants continually readjusted their expectations and approach to dating apps to mitigate negative impacts. This was one of the various coping mechanisms that participants used. In describing how they felt about their dating app use, the following participant explained that they viewed themselves as a successful dating app user largely because of readjusting their expectations:

I’m happy with the interactions overall. I don’t feel like I’m wasting my time or I’m getting frustrated. And I think that’s partially because I have low expectations of what it could be. It’s like, hey, if I chat with someone off and on for a couple of days and then that peters out, that’s fine.

P6

Later in their interview, they explained that, because rejection is common, they delineated between different instances of rejection that did and did not bother them:

On dating apps, I’ve got that lower expectation. Whereas if it is someone that I’ve met organically or subsequently met in person, then that rejection is [impactful because] I’ve invested more time in it. They’ve also had a chance to kind of experience a more authentic me, because in person you’re just going to learn more about a person. And so, then it becomes a more meaningful rejection where it’s like, okay, they actually got to see who I am, we chatted face to face for hours while, we had dinner or something like that. And so now the fact that they’re rejecting me is more significant. I’m not saying it’s devastating, but it’s more significant than if someone suddenly unmatched with me on Tinder or said, “It’s not going to work out.”

P6

Other participants explained that ghosting is distressing because of the uncertainty associated with it. When asked why being ghosted was distressing, one participant explained the following:

Because I don’t know how to [act], because do I stay there to talk to no one? Or do I know who I can talk to? Now, since I’ve had clear rejections or [a] clear closed door...ghosting is [by comparison] unclear. So, to me, maybe that says something about my personality...It makes me anxious. So, I'm like, okay, [did] I do something that is not okay? What is it? Do I need to stop or, do I need to have another strategy? It makes my hamster go on. Whereas someone is telling you no is a no, you can move on.

P12

Overall, participants understood that, because of the nature of online interactions and social media, the impact on their well-being was proportionate to the amount of effort devoted to a particular relationship. One participant summarized the following:

Some interactions, like heavy rejection or something, is kind of like, oh, that sucks...sometimes you do close the app and you feel a little worse about yourself or something. But it’s kind of like, for me, very transient and just moves on, and I go about my day. I think overall, I feel more positive about myself using dating apps. Because if you’re not, I feel like just day to day out at places like, compliments are super rare. Really, having deep conversations with anyone is super rare. So, it’s kind of like, there’s not a lot of places for an ego boost in any way. At least, that’s the sense I get going about shopping or hanging out somewhere. It’s kind of a lot less affirming, in a way, to be out in the world than on a dating app where people are hitting you up because they’re attracted to you or something. And that feels good. And so, I do think that the overall effect of using the app has been positive for me, and that has been—ego boost is not the word I’m looking for, but just something validating almost. Something that makes me feel like, yeah, I am attractive. I’m not wasting my time here. People do message me and stuff. That feels good.

P14

### Dating App Use as a Medium for Community Connection

The mean of the friendship or networking subscale of the Uses and Gratifications of Grindr scale was the lowest (3.95, SD 1.27), suggesting that participants felt neutral about using dating apps to make friends or connect with the queer community. However, most survey participants reported currently using dating apps to make new friends (151/249, 60.6%) compared to when they started using dating apps (114/250, 45.6%). The SEM revealed that the intensity of dating app use was unrelated to community connectedness (β=0.00; *P=*.99); however, using dating apps for friendship or networking was strongly associated with community connectedness (β=0.26; *P*<.001). Community connectedness was, in turn, moderately associated with life satisfaction (β=0.18; *P=*.01) and self-esteem (β=0.13; *P=*.04). That is, the more participants used dating apps for friendship, the greater their reported community connectedness, and the greater the community connectedness, the higher the scores of life satisfaction and self-esteem. These quantitative findings highlight how participants’ intentional use of dating apps can foster alternative and meaningful relationships, which is further explored in the third theme: *opportunities for meaningful community connections on dating apps.*

Interview participants explained how dating apps offer opportunities to connect with other queer people, enriching their lives and improving their well-being. One participant explained the following:

I’m pretty isolated, don’t have many in-person friends, so it’s just a good way for me to communicate with people.

P10

Making friendships online was described as being challenging:

...deeper friendships and that sort of thing, and it’s hard to get that from these apps.

P5

Participants explained that, often, people’s expectations are sexual or romantic, and therefore, friendships emerge as a by-product of other types of relationships that did not form. One participant who was skeptical about being able to make friends remained hopeful, noting the following:

I mean, I don’t genuinely think that I’ll make friends on Grindr. I just frequently, uh, convince myself that there’s a sliver of hope.

P9

While some participants went through periods of using dating apps for friendship (ie, having the motivation for seeking friendships), they explained that they often did not actively use them to make friends but allowed those relationships to form if the opportunity emerged. One participant explained the following:

I’m not particularly seeking out other things. That being said, I do have several friends that I’ve made from Grindr. That’s why I’m open to just talking to lots of people. Even if sex isn’t the first thing on my mind in that moment. Just because I do know that several of them have evolved into good friendships...So, there are other social relationships I go for, [they] just tend to be secondary. Or if they happen coincidentally, it’s a natural fit and that’s welcome. My profile doesn’t say looking for friends, period.

P3

Participants who did make new friends through dating apps felt enriched and satisfied with their expanded queer social circles. In general, dating apps functioned as a convenient queer social space where positive, negative, and neutral interactions occurred. One participant explained the following:

I don’t think I really use [dating apps] for just one thing...I’ve met a lot of great platonic friends on there. I met my partner, who we’re going on four years now on Grindr. Um, I like in the silliest, mundane way, I also, I work at a bathhouse and sometimes, like, communicate with customers on Grindr...I found a roommate on Grindr once...It’s a very all-purpose app for me.

P11

### Dating App Discrimination and Mental Health

Nearly all survey participants (239/250, 95.6%) reported at least one experience of dating app–related discrimination in the previous 12 months. The mean score on the dating app discrimination scale was 2.78 (SD 1.10), indicating that, on average, participants experienced dating app discrimination between a few times a year and a few times a month. However, the SEM revealed that intensity of dating app use was unrelated to dating app discrimination (β=0.11; *P=*.23). Dating app discrimination, conversely, had a small to moderate negative association with life satisfaction (β=−0.15; *P=*.02) and self-esteem (β=−0.17; *P=*.01) but a moderate positive association with depression (β=0.39; *P*<.001) and anxiety (β=0.30; *P*<.001); that is, a higher number of reported experiences of dating app discrimination were associated with worsened mental health. These quantitative findings demonstrate the prevalence and impact of discrimination on dating apps, which is continually resisted by queer adults, as is explored in the final theme: *resisting ongoing discrimination and fetishization on dating apps.*

Most of the interview participants explained that discrimination and fetishization (ie, objectification and exoticization) are an ongoing issue on dating apps that is continually evolving and presenting itself in new ways. A few participants noted that they had not experienced discrimination themselves but acknowledged that other marginalized users likely had. Participants primarily discussed issues about beauty standards, ageism, fatphobia, genderism and transphobia, and racism.

Discrimination online, according to participants, is continually changing. Although it may have been common to see outright racist, fatphobic, or femmephobic rhetoric in the past, discrimination online is now more commonly presenting itself through the rhetoric of “personal preference.” As one participant described, on other users’ profiles, “You might see, ‘Blue Lives Matter.’ You might see ‘no fat, no femmes...Asian.’ You might see...‘only into white guys,’ ‘not into black guys.’ You might see ‘masc’” (P4). Another participant described how they noticed a shift in racist rhetoric following the murder of Mr George Floyd and the subsequent Black Lives Matter organizing in 2020, but these changes did not necessarily apply to other axes of discrimination:

I can literally draw a line at George Floyd between “whites only” and never seeing that anymore. I saw that very common, like, “whites to the front of the line,” “Caucasian only,” stuff like that. Um, so very explicitly racist. I think I’ve seen it once since George Floyd, and it was very common before that, which they either stopped being racist or went into hiding, perhaps more so the second one...Still, some of that again, “trans only” is a very common one or “trans/CD,” I think they mean cross dresser or cross dressing...I see that daily...Vaccinated, like “vaccines only” is definitely one that I encounter...circumcised versus uncircumcised—I’ve definitely seen that on profiles as well like looking for one or the other only.

P3

This participant also remarked on recent reports of COVID-19 vaccinations (and subsequently mpox vaccinations) on users’ profiles—with some apps such as Grindr designating a space on the profile to report vaccination status.

Participants’ experiences of discrimination shaped their overall experience with dating apps. One participant who recently immigrated to Canada explained how their confidence was diminished through experiencing sexual racism, which only occurred once they moved to Canada:

My confidence was shattered initially, and I was feeling that I think it’s happening with me only. And I felt maybe I am not at all good looking, maybe I have lost my charm or something. I don’t know what was happening to me...And then I just tried to change my pictures, look more attractive, uh, flaunt my ABS, flaunt my muscles, something. But then also, things were not working. Things were improving, but not working.

P15

This participant explained that their well-being was especially impacted by the racism they experienced online, which largely manifested as experiencing rejection and lack of interest from others. They went on to explain that, when they discussed their experiences with other racialized friends, they were validated in their experiences and altered their approach to online dating:

So, when I discussed with my friends and they told me this is quite, uh, common here—discrimination—I felt, yeah, it’s okay. So now I have actually stopped approaching such people. I can say, I don’t want to feel this. I can say this is harassment, or, um, put on your dignity. I don’t want to do that. Uh, so I’ve stopped approaching [other users]. If someone approaches me, sure. My first answer to them is that I’m Asian. And if they’re comfortable, then only [will] I share my pictures.

P15

Another participant who experienced fetishizing and sexualized racism as a Black gay man used a different kind of coping mechanism:

I would warn people about sexual racism [on my profile] because that’s something that happened a lot when [previously], and, uh, when I didn’t put something on my profile...when I was younger, uh, let’s say at the beginning of Grindr, I was very shy about those messages, so I wouldn’t talk about them.

P12

This participant explained that including a disclaimer on their profile decrying sexual racism circumvented the need to confront bigoted users. They continued to describe other strategies that helped them navigate these experiences, including journaling and keeping a record of racist interactions:

When I realized that there were a lot of, uh, Black gay men that were having these experiences, I’ve started to do some journaling about it. So, I would do screenshots, uh, of those crazy message...I like the idea to have a personal archive, to also sometimes give yourself a perspective that you may have a lot of these messages for two months, but it’s not the entirety of your experience of the app. It gives you back the feeling of the time passing by. Because I’ve realized that when I’ve had a depression, uh, when you’re depressive, you don’t feel the time passing by, and you don’t have a notion of time. And this archiving process helped me having a perspective about these messages.

P12

Some participants noted that the discriminatory or fetishistic experience affirmed or reaffirmed an identity they had or made them feel better about themselves. For example, one user in his 60s mentioned typically experiencing ageism online but that one experience left him feeling complimented:

There’s one guy he said, I’m looking for a 50-year-old plus. And so, I sent my masked picture, he comes back to me, says, “You’re not old enough,” and I didn’t tell my age. Uh, and he made me smile. Whereas all these other ones, they’re like, “No, you’re too old.” So once in a while, there’d be something that really, uh, brightens my day.

P22

Another participant similarly explained that they were affirmed in their transgender identity when other users were clear about being interested in connecting with transgender women:

I find somewhat liberating the fact that they know what they’re looking for. Most of them, of course. So, if they know what they’re looking for, and they know what to expect, and they look at me and still find some interest to me, well, that’s reaffirming that makes me feel empowered. I don’t want to sound cheesy, of course, but that makes me feel like all this effort I put all those years trying to keep away my negative thoughts about myself are worth it.

P7

## Discussion

### Principal Findings

This mixed methods research explored the experiences of queer adults in Canada regarding dating app use and its association with mental health. Dating app use is associated with some aspects of mental well-being (in specific contexts and times), particularly for users using strategies to improve their experience. Although all the participants in this study were recruited through Grindr, on average, participants reported currently using approximately 3 apps and up to 11, similar to a US study [108]. The queer peoples in our study generally used dating apps several times a day for over a year and with multiple motivations. Over 80% of the sample (208/249, 83.5%) reported that seeking sex or hookups was a current motivation for their dating app use, and roughly half (121/248, 48.8%) noted that sex was a primary goal. Previous literature has documented that queer dating apps are often hypersexualized [109,110]. Nevertheless, our findings revealed that queer peoples use dating apps in messy ways, often adjusting their use to satisfy their present needs. Indeed, participants described how their use was inconsistent; shifting; evolving; and not clearly categorized as looking for sexual, platonic, or romantic relationships. Studies on queer social arrangements have found that casual sex is not always at odds with relationship development [37,111]. Our findings highlight queer adults’ openness to serendipity and surprise, being hopeful that novel interactions may generate different and unexpected relationships.

Given that many dating apps are designed for casual sex and fleeting connections [48,51], queer adults described being able to use dating apps for sex with relative ease. Previous studies have noted that queer dating app users experience frustration with the inability to form meaningful relationships on apps such as Grindr and that this may be a reason for discontinuing use [51], whereas our findings provide the additional nuance that queer peoples acknowledge that long-term relationships require effort and are more challenging. Rather than discontinuing use entirely, queer peoples may take breaks from dating app use or adjust or readjust their expectations to reduce frustration and disappointment.

Regardless of whether dating app profiles are arranged on a grid (eg, Grindr) or a card stack (eg, Tinder) [112], they create the illusion of an endless supply of potential connections [109]. This is true to a certain extent as users could ostensibly connect with any number of other users. An advantage of dating apps is that they increase people’s choices of potential partners [113]. Companies may restrict connections behind paywalls and premium features to encourage users to pay to expand their reach. Grindr advertises their premium feature, XTRA, as follows: “It unlocks a bunch of features and allows you to connect with others more easily” [114]. The queer adults in this study explained how this seemingly endless supply of new connections contributes to the addictiveness of dating apps. Similarly, gay men have previously described Grindr as addictive [7]. Despite this, queer peoples simultaneously described dating apps as extremely convenient, fitting their schedules and offering them access to a queer “world.”

Queer peoples described dating app practices such as ghosting, ignoring, blocking, and rejection as contributing to frustration and isolation. These exclusionary communication practices are common on dating apps [115]. Previous studies have noted their negative impact on online dating cultures [108]. Although the queer peoples in this study explained how these practices can be negative, they were simultaneously strategies used to avoid unsafe or unwanted interactions. Young Black men who have sex with men have similarly used blocking as a strategy to avoid interacting with racist users [116]. In this way, queer peoples negotiate and renegotiate the use of otherwise distressing features of dating apps to improve their personal experience.

Unlike our hypothesized model, the final SEM did not include overall mental health as a second-order latent variable due to poorer model fit. This approach meant that we viewed the mental health indicators as being conceptually separate (eg, self-esteem as distinct from symptoms of psychological distress) and that dating app use affects each of these mental health indicators independently; of course, the model also revealed substantial covariance between the mental health indicators used. While this approach means that we cannot discuss how using dating apps is associated with “mental health” as a general construct, we describe the specific pathways in the following paragraphs. This elucidates distinct associations between dating app use and mental health measures, reflecting the nuanced ways in which digital sociality is experienced. These differences reinforce our reparative and transformative framework, emphasizing lived complexity over reductive models of harm. Furthermore, the qualitative findings support the distinctiveness of mental health components. For instance, participants described how dating apps could simultaneously elevate self-esteem through affirmation while also triggering anxiety or disillusionment. This supports our decision to preserve separate outcome pathways and reject a second-order latent variable as collapsing these constructs risks erasing the contradictory and temporal aspects of users’ emotional experiences.

This study found that dating app use intensity was not related to symptoms of depression or anxiety among queer adults. Instead, the increased intensity of use was associated with greater life satisfaction and self-esteem. Our findings did not show support for hypothesis 1 and are inconsistent with those of previous literature showing that excessive social media use is associated with increased psychological distress and less life satisfaction [5,39-43]. A recent study among Portuguese students in higher education found no difference in depression scores among dating app users versus nonusers but higher depression and anxiety scores among gay or bisexual participants [4]. Nearly half (113/248, 45.6%) of queer adults in our study reported a depression diagnosis, and over a third (95/248, 38.3%) reported an anxiety diagnosis. Although we could not to compare mental health outcomes between dating app users and nonusers, qualitative accounts support our findings. Queer adults used dating apps with intention and conscientiously, adjusting their use and coping mechanisms to mitigate the adverse outcomes. For the queer adults in our study, dating apps were simply another tool for social connection, which may facilitate (sometimes simultaneously) distressing or pleasant interactions and experiences. The mundane and routinized description of dating app use demonstrates how virtual sociosexual spaces have become enmeshed with queer peoples’ everyday lives.

The final SEM that was interpreted did not include interaction terms to assess whether dating app use motivations moderated the association between dating app use and mental health. As such, we are unable to directly answer hypothesis 2; instead, we discuss the main effects of different motivations on mental health. Using dating apps for sex was unrelated to all mental health outcomes, showing some support for hypothesis 2a that using dating apps for sex was not associated with poorer mental health. However, our finding is contrary to those of previous literature showing that using dating apps for sex was associated with higher self-esteem and life satisfaction among men who have sex with men [43]. Using dating apps for romance was negatively associated with life satisfaction, supporting hypothesis 2b. While dating apps continue to be hypersexualized spaces suited to foster casual sex [7,53], our findings suggest that queer peoples are continuing to use dating apps for romantic pursuits with little to no negative mental health implications. Some qualitative accounts indicated that queer adults are nevertheless disappointed and negatively impacted by the fleeting nature of connections that is typical on dating apps. As this cross-sectional study was correlational, whether using dating apps for romance *caused* lower life satisfaction cannot be determined; it may be that queer adults with lower life satisfaction turn to dating apps to find romantic partners. For example, being married was associated with greater life satisfaction in a study with a national sample of adults in the United States, including queer peoples [117].

Using dating apps for social approval was associated with poorer mental health, supporting hypothesis 2c and consistent with previous literature showing that using dating apps for self-esteem enhancement was associated with problematic Tinder use [56]. One study that did not report sexual identity found that, although Tinder users reported higher negative affect, there was no difference between the self-esteem and depression scores of Tinder users versus nonusers [118]. Dating apps may function to both boost and hinder one’s self-esteem [56,119]. Low self-esteem may lead to pursuing external validation [120]. Qualitative accounts similarly described ways in which online interactions may validate and affirm or reaffirm personal identities and bolster self-worth. A young transgender woman likened seeking external validation to addiction, noting initial pleasure but later negative feelings. Her account further complicates the relationship between using apps for external validation and mental health, suggesting that their association may vary with time and context (including social, cultural, and financial resource availability). Further research, including longitudinal studies, is needed to explore how the relationship between dating app use and mental health varies over time.

Dating app use was unrelated to queer community connectedness, thus disproving hypothesis 3 that dating app use is negatively associated with community connection. However, using dating apps for friendship was related to community connectedness, supporting hypothesis 4. As predicted, community connection was strongly associated with life satisfaction and self-esteem. Queer peoples shared successful friendships resulting from their dating app use, highlighting how they had enriched their lives, and it was associated with greater perceptions of queer community connectedness. Making friends online was often not a primary motivation for use. Queer peoples nevertheless welcomed serendipitous friendships and community connections. For one participant, friendships were the most common outcome of their app use despite being their lowest priority. Queer intimacy and friendships are intertwined on dating apps [121]; online interactions can generate friendships and foster unexpected relationships. Zervoulis et al [43] argued that, although their finding was nonsignificant, the less queer men used dating apps, the greater their sense of community and vice versa. Our findings indicate that dating apps provide queer peoples access to queer communities, especially when they are actively seeking to form new friendships.

Discrimination persists on dating apps, with nearly all queer adults in this study (239/250, 95.6%) reporting at least one experience. Our findings revealed that dating app use was unrelated to experiences of discrimination online, failing to show support for hypothesis 5 that dating app use is positively related to discrimination. Discrimination online is likely so pervasive that participants reported a perceived experience of discrimination regardless of how intensely they used dating apps. Physical appearance was the most common reason attributed for experiencing discrimination. Dating apps prioritize self-presentation [109], primarily through photos, and therefore, it is unsurprising that dating app users filter and select others based on appearance. Scholars have discussed a new form of stigma coinciding with dating apps, which manifests through communication practices (eg, blocking) and under the guise of personal preference [109,122,123]. However, participant accounts suggest that, while pervasive, this stigma is changing. This was particularly the case for racialized sexual discrimination, which has become more covert following Black Lives Matter advocacy in 2020. Mowlabocus [124] has similarly argued that Grindr’s Kindr initiative, launched in 2018, was a performative attempt to curb racism on the platform by leveraging politeness politics to maintain the status quo of discrimination through blocking, exclusion, filtering, and ignoring as opposed to outright racism. Our findings further reveal how sexualized racism has become covert by appealing to politeness and personal preference.

As hypothesized, experiencing dating app discrimination was associated with poorer mental health, including increased anxiety and depression and decreased life satisfaction and self-esteem. However, queer peoples used various strategies to mitigate the harms associated with experiencing discrimination, including relying on social networks, decrying discrimination on their profiles, and keeping a record of these experiences. Distress disclosure has previously been shown to be a socially conducive strategy and can lower psychological distress [125]. Moreover, keeping an archive of discriminatory social interaction has previously been noted as a resistance and refusal strategy. Douchebags of Grindr was a blog showcasing screenshots of discriminatory Grindr users, creating an archive to contest individual preferences that justified discrimination [126]. This counterarchiving practice shamed men for discriminatory practices and became a space where individuals resisted the discriminatory norms on Grindr. Queer peoples are actively engaging in resistance and refusal to survive the threatening online space both through actions on Grindr and by creating alternative spaces. It is important to underscore that queer individuals often do not want to engage with bigoted users. Either through proclamations in their profile descriptions or by concealing a stigmatized identity, queer peoples are using strategies to dissuade negative interactions. Queer individuals concealing their identity are claiming and reclaiming the ability to make their identities visible or invisible by having greater control over when they disclose their identity and to whom.

### Implications for Public Health

The queer adults in this study discussed the innovative, important, and effective strategies that they used to prevent the harms associated with dating app use. Some of the data from this research suggest that interventions may not be necessary in many cases. Queer peoples are strong and resourceful, continually working to fulfill their needs. While it is important not to overstate the findings of this research alone, it is our hope that this work has highlighted the diverse abilities of queer peoples to navigate the digital world through resources and strategies for them by them. We briefly describe implications for public health in this section; a comprehensive discussion of the public health implications of this work has been reported elsewhere [62].

Health and educational resources should be made available to queer peoples to use if needed. While many adults in this research used successful coping mechanisms, they collectively described feelings of loneliness and isolation as well as the need to feel affirmed and validated by others regarding their dating app experiences. One such strategy would be to make available public health or community-led educational interventions regarding dating app experiences and strategies to navigate the online space (including safety strategies, setting realistic expectations, and health promotion strategies). The information regarding these resources could be disseminated through advertisements on dating apps (eg, Grindr Ads), social media accounts of dating apps (eg, Grindr’s Instagram page), queer community forums (eg, queer social media pages), and community organizations and queer health clinics. Previous literature has reported the effectiveness of implementing sexual health promotion and educational strategies on dating apps [127,128]. These strategies can be expanded to include mental health, self-efficacy, and behavioral interventions. Although the effectiveness of such strategies remains unknown, a recent study demonstrated the effectiveness of eHealth interventions in a national sample of queer youth (aged 13-18 years) across the United States to use safety strategies when online dating [129]. Educational interventions and resources have the potential to provide queer dating app users with the skills and insights to mitigate the harmful effects of dating app use.

### Strengths and Limitations

To our knowledge, this research is the first to use mixed methods to explore the association between dating apps and mental health among queer adults across Canada. Participants represented diverse identities and experiences. The high completion rate and member check enhanced our interpretations and improved the relevance of the findings to the queer peoples involved in this research. Although this research was not interested in making broad claims about generalized experiences for all queer peoples, our findings contribute to the literature interrogating power relations on dating apps and their relationship with well-being. To be clear, this research was interested in interrogating power relations on dating apps, highlighting how power is continually resisted and refuted through the everyday, the mundane, and the unintentional. Our sample, recruited primarily through Grindr, comprised predominantly cisgender gay men; the sample nevertheless represented substantial diversity of ages, incomes, ethno-racial identities, relationship statuses, and locations in Canada (including rurality), among others. However, we emphasize that it was not the intention of this work to describe the experiences of a particular made-marginalized queer identity per se (eg, White gay men) but, rather, to interrogate how axes of power shape experience and well-being online (eg, resisting transphobia as a transgender or cisgender person). In this way, the findings describe how motivations and intentions of use and experiences online (eg, discrimination) are related to well-being while centering reparation. While these findings do not necessarily generalize to all intersections of queer peoples who use dating apps—nor was this our aim—they elucidate the manifestations of power relations in virtual sociosexual spaces.

This research is not without limitations. Participants were recruited exclusively through Grindr, a gay dating app predominantly for cisgender men, resulting in a sample that reflected this demographic. Although the maximum variation recruitment approach centered the voices of other queer peoples, there was a notable underrepresentation of transgender men and individuals assigned female at birth. Moreover, we may not have captured the unique experiences of queer peoples who pay for premium features on Grindr (and, thus, do not receive advertisements) or those who use other dating apps exclusively but not Grindr. Future research may use diverse recruitment strategies to further diversify their sample, including advertising on multiple dating apps (eg, Tinder and Lex) and other social media platforms (eg, Instagram and Facebook). Other successful recruitment strategies may include recruiting through queer community organizations, sexual health clinics, and word of mouth.

Our sample may also primarily comprise queer adults who have successfully navigated dating apps, whereas those who have been drastically negatively impacted may have discontinued use entirely. Two-thirds of the sample (164/248, 66.1%) also reported at least one mental health diagnosis, perhaps indicating that the queer adults we engaged were already connected to mental health supports or implementing strategies to promote their mental health. While the recruitment process was rigorous, requiring an eligibility call to confirm participants’ identities may have been a barrier to participation for some.

Due to the correlational study design, we could not determine the causal nature or direction of the associations uncovered. A history of mental illness is a strong predictor of future mental health; this, along with other covariates such as social location, was not captured in our analyses. However, the integration of quantitative and qualitative strands further supported our findings. Finally, some survey items included suggested responses, which may have guided participants to think only about the options presented (and not others).

### Conclusions

This research critically explored queer peoples’ dating app use and associated mental health using reparative theory. Dating app use intensity is positively associated with some aspects of mental well-being, especially for queer adults who use strategies to improve their experience. Queer peoples use dating apps in a routinized and mundane way, indicating how the virtual sociosexual space has become enmeshed with their daily lives. Dating apps offer queer peoples access to community, connection, and romance. Although the space is primarily sexual, intimacy online can and does result in other types of relationships. Queer adults leverage hope and serendipity to stumble upon new and welcomed connections. Although experiencing dating app discrimination and seeking social approval were associated with some indicators of diminished mental health, queer peoples described using various coping mechanisms to protect themselves and promote their well-being. Overall, queer peoples use dating apps messily and differently, in ways that are continually changing. The relationship between dating app use and well-being is context dependent and temporally dynamic. This research has contributed to our understanding of how queer peoples collect the necessary resources to navigate the virtual sociosexual space.

## Appendix

Multimedia Appendix 1 Checklist for Reporting Results of Internet E-Surveys (CHERRIES).

Multimedia Appendix 2 Sociodemographic and mental health characteristics of the survey respondents (N=250).

Multimedia Appendix 3 Dating app use characteristics of the survey respondents (N=250).

## Abbreviations

COREQ: Consolidated Criteria for Reporting Qualitative Research
REDCap: Research Electronic Data Capture
RMSEA: root mean square error of approximation
SEM: structural equation model

## Data Availability

The datasets generated or analyzed during this study are not publicly available due ethics and privacy limitations but are available from the corresponding author on reasonable request. Qualitative data are not available.
