# Supplementary material for: Depathologizing Queer Adults’ Dating App Use in Canada: Convergent Mixed Methods Study
Source: J Med Internet Res. 2025 Jul 23;27:e72452. doi: 10.2196/72452 (PMC12329389; doi:10.2196/72452)
Supplement: Multimedia Appendix 1 [file jmir_v27i1e72452_app1.pdf]

Multimedia Appendix 1. *Cherries Checklist*

**Item Category**<br>Checklist Item	**Explanation**
**Design**	
Describe survey design	This research sought to recruit queer adults (18+) who use dating apps. A convenience sample was recruited through advertisements on Grindr. The sampling strategy was purposive sampling with maximum variation. The full recruitment process is described in the methods.
**IRB (Institutional Review Board) approval and informed consent process**	
IRB approval	Research Ethics Approval was sought from the University of Toronto Research Ethics Board
Informed consent	After meeting with the lead researcher to confirm eligibility, participants received a unique link to complete the survey. The beginning of the survey included an informed consent form specific to the online survey portion of the research. Participants were told the estimated length of time of the survey, how the data would be stored and for how long (de-identified data for up to 7 years), who the lead investigator and research team were, and the purpose of the research. Participants were also told about the benefits and risks, compensation, and how to withdraw data within 1 week of completing the study.
Data protection	Participants were told about safety processes to secure their personal data. The language in the consent form was as follows: Your participation in this research will be known only to the research team. The information that you provide to us will be kept confidential. Only the research team at the University of Toronto will have access to this information. The people who work with us have an obligation to keep all research information confidential. All your identifying information (such as your name and contact information) will be securely stored separately from your research information. We will use a participant number (not your name) in our written and computer records so that the research information we have about you contains no names. During the study, all electronic records will be kept secure in an encrypted file on the lead researchers' password-protected computers. Any non-identifiable datasets will be stored on the University of Toronto's One Drive, which is a secure platform.<br><br>We will describe and share our findings in reports to the community as well as journal articles and presentations. We will only report group results and not individual results. This means that you will not be identified in any way in our reports.<br><br>The research team will use the University of Toronto's One Drive to share documents between team members for the purpose of data

	analysis and report writing. This platform is secure, encrypted, and stored in Canada.
**Development and pre-testing**	
Development and testing	The survey was developed by the lead researcher in Qualtrics and the survey was piloted with 10 queer adults in the research team's network. The pilot assessed estimated time to completion, comprehension of questions/survey, useability and technical functionality.
**Recruitment process and description of the sample having access to the questionnaire**	
Open survey versus closed survey	The survey was closed, where each participant required a unique link, which was emailed to them, to complete the survey. Using this link, participants could complete the survey in more than one setting (i.e., saving their responses and returning to complete the survey at a later time).
Contact mode	Participants were recruited through Grindr, who saw an advertisement and were directed to complete an intake survey to express interest. Prospective participants were invited to a brief zoom call to confirm eligibility and be assigned to the quantitative and/or qualitative phases of the study. Participants were emailed a link to complete the online survey.
Advertising the survey	The research study was advertised on Grindr.
**Survey administration**	
Web/E-mail	The survey was hosted on Qualtrics. A unique access link was emailed to participants directly through the Qualtrics platform.
Context	The research study was advertised on Grindr, a dating app for queer adults (18+) largely used by gay, bisexual, and queer men. The advertisement was displayed to a random selection of users who were active (i.e., online at the time of the advertising period) and were currently located in Canada.
Mandatory/voluntary	The intake survey was voluntary. Participants who met with the lead researcher were then invited to complete the main study survey on Qualtrics, which was also voluntary. Participants could skip any questions they did not want to answer and withdraw their participation/consent within 1 week of completing the survey.
Incentives	Participants were compensated $10 CAD for completing the survey.
Time/Date	The advertising period on Grindr was between October 25th, 2022, to February 4th, 2023. The data collection period was between November 1st, 2022, to February 20th, 2023.
Randomization of items or questionnaires	Items within scales (i.e., a single questionnaire) were randomized.
Adaptive questioning	Adaptive questioning was used to display a certain set of questions to participants based on previous responses.
Number of Items	The number of items per page varied per survey section and block. E.g., if responding to a question caused the survey to branch, then no other questions followed on that one screen. If the question

	was the final question in a block, it too was the last question on the screen.
Number of screens (pages)	The number of screens/pages also varied according to participant responses (as questions were adaptive). Participants could see a progress bar at the top of the screen which was an indicator of how far along the survey they were.
Completeness check	A completeness check was assessed using Qualtrics (this was a feature built into the online survey platform), checking to make sure that participants reached the end of the online survey, and reviewing 3 attention checks, which were placed throughout the survey.
Review step	Participants were able to go back to change responses that they provided. Participants were not provided a summary of their responses at the end to review.
**Response rates**	
Unique site visitor	Response rates were calculated based on the unique number of individuals who attended an eligibility call and were emailed a unique survey link.
View rate (Ratio of unique survey visitors/unique site visitors)	A view rate cannot be calculated as we are unable to determine the unique number of visitors to the intake survey or those who engaged with the Grindr advertisement.
Participation rate (Ratio of unique visitors who agreed to participate/unique first survey page visitors)	A participation rate cannot be calculated as we are unable to determine the unique number of first survey page visitors (for the intake survey). Based on the unique number of individuals who were invited to the main Qualtrics survey, the participation rate is 94.2%. A total of 278 individuals were invited to the Qualtrics survey, 15 of which did not consent and 1 duplicate (i.e., 262 users agreed to participate).
Completion rate (Ratio of users who finished the survey/users who agreed to participate)	The completion rate was calculated at 97.7%. There was a total of 262 participants who completed the first page, and 256 participants reached the end of the survey. Note – 6 individuals failed to answer 2/3 attention checks properly and were removed from the final analytic sample.
**Preventing multiple entries from the same individual**	
Cookies used	Cookies were not used to assign a unique ID. A unique survey link was emailed to participants after the lead researcher met with them to confirm eligibility.
IP check	IP address was collected as a secondary check to confirm unique survey responses. A unique survey link was emailed to participants after the lead researcher met with them to confirm eligibility. One duplicate was identified and removed.
Log file analysis	Not applicable.
Registration	Prospective participants who saw the Grindr advertisement and were interested in the survey were routed to an intake survey to express their interest. The lead researcher invited prospective participants to a zoom call to confirm their eligibility. Each eligible

	person was emailed a unique survey link to their email to access the Qualtrics Survey (main study survey)
**Analysis**	
Handling of incomplete questionnaires	All responses that moved beyond the demographic portion of the survey (section 1) were analyzed. In the main text/analyses, where n's varied between questions, this was reported.
Questionnaires submitted with an atypical timestamp	Not applicable. The research team used 3 attention checks throughout the survey to assess attention, and participants that failed 2/3 checks were excluded from the analytic sample.
Statistical correction	Not applicable.
