# Supplementary material for: Depathologizing Queer Adults’ Dating App Use in Canada: Convergent Mixed Methods Study
Source: J Med Internet Res. 2025 Jul 23;27:e72452. doi: 10.2196/72452 (PMC12329389; doi:10.2196/72452)
Supplement: Multimedia Appendix 2 [file jmir_v27i1e72452_app2.pdf]

**Multimedia Appendix 2.** *Sociodemographic and mental health characteristics of the survey respondents (N=250).*

| Characteristic | Values |
|---|---|
| **Age (y), n (%)[a]** | |
| 18-24 | 36 (14.4) |
| 25-34 | 103 (41.2) |
| 35-44 | 53 (21.2) |
| >45 | 58 (23.2) |
| **Sex at birth, n (%)** | |
| Female | 6 (2.4) |
| Male | 242 (96.8) |
| Intersex | 1 (0.4) |
| Other | 1 (0.4) |
| **Gender identity, n (%)[a]** | |
| Cisgender man | 200 (80) |
| Transgender man | 1 (0.4) |
| Transgender woman | 13 (5.2) |
| Queer, nonbinary, or other | 36 (14.4) |
| **Sexual orientation, n (%)[a]** | |
| Gay | 167 (66.8) |
| Bisexual or pansexual | 58 (23.2) |
| Queer | 23 (9.2) |
| Straight | 1 (0.4) |
| Lesbian | 1 (0.4) |
| **Relationship status, n (%)[b]** | |
| Single | 170 (68) |
| Legally married | 20 (8) |
| Divorced | 10 (4) |
| Common-law partnership | 18 (7.2) |
| Relationship—>2 y | 19 (7.6) |
| Relationship—<2 y | 17 (6.8) |
| Polyamorous relationships | 17 (6.8) |
| Other | 9 (3.6) |
| Prefer not to answer | 1 (0.4) |
| **HIV status, n (%)** | |
| HIV negative | 228 (91.2) |
| HIV positive | 15 (6) |
| Unknown HIV status | 5 (2) |
| Prefer not to answer | 2 (0.8) |
| **Citizenship status, n (%)** | |

| | |
|---|---|
| Canadian citizen | 206 (82.4) |
| Permanent resident | 20 (8) |
| Temporary resident | 13 (5.2) |
| Nonresident | 4 (1.6) |
| Other | 4 (1.6) |
| Prefer not to answer | 3 (1.2) |
| **Ancestry, n (%)[b]** | |
| Indigenous[c] | 14 (5.6) |
| West African | 4 (1.6) |
| North African | 4 (1.6) |
| East African | 1 (0.4) |
| Caribbean | 7 (2.8) |
| West Asian | 12 (4.8) |
| South Asian | 25 (10) |
| East Asian | 23 (9.2) |
| South or Central American | 24 (9.6) |
| British Isles—European | 81 (32.4) |
| France—European | 28 (11.2) |
| Western European | 33 (13.2) |
| Northern European | 9 (3.6) |
| Eastern European | 24 (9.6) |
| Southern European | 17 (6.8) |
| Jewish | 5 (2) |
| Other | 22 (8.8) |
| Do not know | 5 (2) |
| Prefer not to answer | 3 (1.2) |
| **Educational level, n (%)[b]** | |
| Did not complete high school | 2 (0.8) |
| High school diploma or GED[d] | 75 (30) |
| Currently in or did not complete postsecondary education | 47 (18.8) |
| College certificate or diploma (<4 y) | 43 (17.2) |
| College degree (4 y) | 13 (5.2) |
| Bachelor’s degree | 93 (37.2) |
| Master’s degree | 55 (22) |
| Doctoral degree | 6 (2.4) |
| Professional degree | 14 (5.6) |
| Other | 4 (1.6) |
| Prefer not to answer | 1 (0.4) |
| **Employment status, n (%)[b]** | |
| Employed full time | 128 (51.2) |

| | |
|---|---|
| Employed part time | 47 (18.8) |
| Volunteer part time | 22 (8.8) |
| Seasonal worker | 5 (2) |
| Not working (on disability) | 13 (5.2) |
| Retired | 11 (4.4) |
| Student—postsecondary education | 52 (20.8) |
| Student—high school or equivalent | 1 (0.4) |
| Student—other | 5 (2) |
| Unemployed | 18 (7.2) |
| Looking for work | 21 (8.4) |
| Other | 22 (8.8) |
| Prefer not to answer | 2 (0.8) |
| **Personal income (2021), n (%)** | |
| No income | 15 (6) |
| CAD $1-$49,999 (US $0.73-$36,340.20) | 113 (45.2) |
| CAD $50,000-$99,999 (US $36,340.90-$72,681.10) | 80 (32) |
| CAD $100,000-$149,999 (US $72,681.80-$109,022) | 17 (6.8) |
| ≥CAD $150,000 (US $109,023) | 7 (2.8) |
| Prefer not to answer | 18 (7.2) |
| **Household income (2021), n (%)** | |
| No income | 5 (2) |
| CAD $1-$49,999 (US $0.73-$36,340.20) | 82 (32.8) |
| CAD $50,000-$99,999 (US $36,340.90-$72,681.10) | 81 (32.4) |
| CAD $100,000-$149,999 (US $72,681.80-$109,022) | 33 (13.2) |
| ≥CAD $150,000 (US $109,023) | 20 (8) |
| Prefer not to answer | 29 (11.6) |
| **Location, n (%)** | |
| Atlantic Canada | 19 (7.6) |
| Quebec | 26 (10.4) |
| Ontario | 117 (46.8) |
| The Prairies | 8 (3.2) |
| Alberta | 33 (13.2) |
| British Columbia | 45 (18) |
| Territories | 2 (0.8) |
| **Population of town or city, n (%)** | |
| 0-9999 | 4 (1.6) |
| 10,000-49,999 | 18 (7.2) |
| 50,000-199,999 | 42 (16.8) |
| 200,000-499,999 | 38 (15.2) |
| 500,000-1,499,999 | 75 (30) |

| | |
|---|---|
| ≥1,500,000 | 73 (29.2) |
| **Self-rated mental health, n (%)** | |
| Poor | 37 (14.8) |
| Fair | 84 (33.6) |
| Good | 78 (31.2) |
| Very good | 46 (18.4) |
| Excellent | 5 (2) |
| **Self-reported mental health diagnosis (lifetime; n=248), n (%)[b]** | |
| No mental health diagnosis | 84 (33.9) |
| Anxiety disorder | 95 (38.3) |
| ADD[e] or ADHD[f] | 54 (21.8) |
| Bipolar disorder | 11 (4.4) |
| Depression | 113 (45.6) |
| Learning disability | 15 (6) |
| Substance use disorder | 14 (5.6) |
| Other mental health disorder | 36 (14.5) |
| Other cognitive or intellectual disability | 9 (3.6) |
| **Age (y), mean (SD; range)** | 36.18 (12.39; 18-71) |
| **Financial well-being (score of 1-5), mean (SD)** | 3.02 (0.90)[g] |
| **Self-rated mental health (score of 1-5), mean (SD)** | 2.59 (1.02) |
| **Community connectedness (score of 0-4), mean (SD)** | 2.88 (0.58)[h] |
| **Life satisfaction (score of 1-7), mean (SD)** | 3.98 (1.52)[i] |
| **Depression (score of 1-4), mean (SD)** | 2.24 (0.68)[j] |
| **Anxiety (n=249; score of 1-5), mean (SD)** | 2.65 (1.00)[k] |
| **Self-esteem (score of 1-4), mean (SD)** | 2.80 (0.63)[l] |

[a]Participant responses were free form but were recoded for the study.
[b]Participants could select more than one option.
[c]Indigenous groups in Canada (eg, First Nations, Métis, and Inuit).
[d]GED: General Educational Development.
[e]ADD: attention-deficit disorder.
[f]ADHD: attention-deficit/hyperactivity disorder.
[g]Cronbach α=0.79.
[h]Cronbach α=0.88.
[i]Cronbach α=0.9.
[j]Cronbach α=0.86.
[k]Cronbach α=0.95.
[l]Cronbach α=0.91.
