# Supplementary material for: Depathologizing Queer Adults’ Dating App Use in Canada: Convergent Mixed Methods Study
Source: J Med Internet Res. 2025 Jul 23;27:e72452. doi: 10.2196/72452 (PMC12329389; doi:10.2196/72452)
Supplement: Multimedia Appendix 3 [file jmir_v27i1e72452_app3.pdf]

**Multimedia Appendix 3.** *Dating app use characteristics of the survey respondents (N=250).*

| Variable | Values |
|---|---|
| **Dating apps used, n (%)[a]** | |
| Grindr | 248 (99.2) |
| Tinder | 120 (48) |
| Scruff | 95 (38) |
| Bumble | 58 (23.2) |
| Hinge | 55 (22) |
| Squirt | 52 (20.8) |
| BBRT | 19 (7.6) |
| OkCupid | 17 (6.8) |
| Sniffies | 17 (6.8) |
| GROWLr | 16 (6.4) |
| Taimi | 13 (5.2) |
| Plenty of Fish | 12 (4.8) |
| Hornet | 9 (3.6) |
| Jack'd | 9 (3.6) |
| Manhunt | 9 (3.6) |
| Badoo | 5 (2) |
| Coffee Meets Bagel | 4 (1.6) |
| Her | 4 (1.6) |
| Happn | 3 (1.2) |
| Match.com | 3 (1.2) |
| eHarmony | 2 (0.8) |
| Other | 35 (14) |
| **Frequency of app use, n (%)** | |
| ≥5 times a day | 121 (48.4) |
| More than once a day but <5 times a day | 69 (27.6) |
| Once a day | 19 (7.6) |
| A few days a week | 34 (13.6) |
| Approximately once a week | 4 (1.6) |
| Less than once a week | 3 (1.2) |
| **Duration of app use (n=249), n (%)** | |
| <1 mo | 2 (0.8) |
| >1 mo but <6 mo | 19 (7.6) |
| >6 mo but <1 y | 13 (5.2) |
| >1 y | 215 (86.3) |
| **Initial reasons for app use, n (%)[a]** | |
| To make new friends | 114 (45.6) |
| To meet people to have sex with | 185 (74) |

| | |
|---|---|
| To find someone to date | 155 (62) |
| To kill time | 80 (32) |
| To connect with the queer community | 89 (35.6) |
| Other | 10 (4) |
| **Current reasons for app use (n=249), n (%)[a]** | |
| To make new friends | 151 (60.6) |
| To meet people to have sex with | 208 (83.5) |
| To find someone to date | 138 (55.4) |
| To kill time | 114 (45.8) |
| To connect with the queer community | 98 (39.4) |
| Other | 10 (4) |
| **Initial top reason for app use (n=243), n (%)** | |
| To make new friends | 20 (8.2) |
| To meet people to have sex with | 119 (49) |
| To find someone to date | 76 (31.3) |
| To kill time | 8 (3.3) |
| To connect with the queer community | 15 (6.2) |
| Other | 5 (2.1) |
| **Current top reason for app use (n=248), n (%)** | |
| To make new friends | 30 (12.1) |
| To meet people to have sex with | 121 (48.8) |
| To find someone to date | 58 (23.4) |
| To kill time | 24 (9.7) |
| To connect with the queer community | 9 (3.6) |
| Other | 6 (2.4) |
| **Experienced dating app discrimination, n (%)** | 239 (95.6) |
| **Perceived reasons for dating app discrimination (n=237), n (%)[a]** | |
| HIV status | 8 (3.4) |
| Ethno-racial identity | 79 (33.3) |
| Age | 107 (45.1) |
| Weight | 75 (31.6) |
| Sexual position—bottom | 34 (14.3) |
| Sexual position—versatile | 19 (8) |
| Sexual position—top | 11 (4.6) |
| Religion | 5 (2.1) |
| Sexual orientation | 32 (13.5) |
| Gender identity | 40 (16.9) |
| Gender expression—feminine | 43 (18.1) |
| Gender expression—masculine | 16 (6.8) |
| Perceived immigration status | 28 (11.8) |

| | |
|---|---|
| A disability | 8 (3.4) |
| Something else related to their physical appearance | 88 (37.1) |
| Income | 23 (9.7) |
| A mental health issue | 25 (10.5) |
| Other | 58 (24.5) |
| Prefer not to answer | 5 (2.1) |
| **Intensity of dating app use (score of 1-5), mean (SD)** | 3.17 (0.72)[b] |
| **Uses and Gratifications of Grindr scale (score of 1-7), mean (SD)[c]** | |
| Social inclusion or approval | 4.25 (1.52)[c] |
| Sex | 5.11 (1.35)[d] |
| Friendship or networking | 3.95 (1.27)[e] |
| Entertainment | 4.73 (1.28)[f] |
| Romantic relationships | 4.22 (3.51)[c] |
| Location-based searching | 4.49 (1.84)[g] |
| Dating app discrimination (score of 1-6), mean (SD) | 2.78 (1.10)[d] |

[a]Participants could select more than one option.

[b]Cronbach α=0.71.

[c]Cronbach α=0.89.

[d]Cronbach α=0.88.

[e]Cronbach α=0.80.

[f]Cronbach α=0.61.

[g]Cronbach α=0.79.
